# Drug resistance emergence in macaques administered cabotegravir long-acting for pre-exposure prophylaxis during acute SHIV infection

**DOI:** 10.1038/s41467-019-10047-w

**Published:** 2019-05-01

**Authors:** Jessica Radzio-Basu, Olivia Council, Mian-er Cong, Susan Ruone, Alicia Newton, Xierong Wei, James Mitchell, Shanon Ellis, Christos J. Petropoulos, Wei Huang, William Spreen, Walid Heneine, J. Gerardo García-Lerma

**Affiliations:** 1 Laboratory Branch, Division of HIV/AIDS Prevention, National Center for HIV/AIDS, Viral Hepatitis, STD, and TB Prevention, Centers for Disease Control and Prevention, 1600 Clifton Road, Atlanta, GA 30329 USA; 2 Monogram Biosciences, 345 Oyster Point Blvd, San Francisco, CA 94080 USA; 3 ViiV Healthcare, Research Triangle Park, NC 27709 USA

**Keywords:** Antiviral agents, HIV infections

## Abstract

A long-acting injectable formulation of the HIV integrase inhibitor cabotegravir (CAB-LA) is currently in clinical development for PrEP. Although the long plasma half-life of CAB-LA is an important attribute for PrEP, it also raises concerns about drug resistance emergence if someone becomes infected with HIV, or if PrEP is initiated during undiagnosed acute infection. Here we use a macaque model of SHIV infection to model risks of drug resistance to CAB-LA PrEP. Six macaques infected with SHIV received CAB-LA before seroconversion. We show integrase mutations G118R, E92G/Q, or G140R in plasma from 3/6 macaques as early as day 57, and identify G118R and E92Q in viruses from vaginal and rectal fluids. G118R and G140R confer > 800-fold resistance to CAB and cross-resistance to all licensed integrase inhibitors. Our results emphasize the need for appropriate HIV testing strategies before and possibly shortly after initiating CAB LA PrEP to exclude acute infection.

## Abstract (web summary)

Long-acting formulation of the integrase inhibitor cabotegravir (CAB LA) is in clinical development for HIV pre-exposure prophylaxis (PrEP). Here, using a SHIV macaque model, the authors show emergence of integrase mutations associated to CAB LA PrEP that confer pan-integrase inhibitor resistance.

## Introduction

With ~36.7 million people living with HIV at the end of 2016 and 1.8 million people becoming newly infected in 2016, the HIV epidemic continues to be a significant public health concern^1^. Daily pre-exposure prophylaxis (PrEP) with the combination of oral emtricitabine (FTC) and tenofovir disoproxil fumarate (TDF) is highly effective in preventing HIV acquisition among men and women (reviewed in ref. ^2^). However, many people find it difficult to adhere to the daily oral regimen and, therefore, cannot fully benefit from PrEP. Long-acting antiretroviral drug formulations that require infrequent dosing and can provide long-lasting protection have the potential to improve adherence and maximize PrEP effectiveness.

Cabotegravir (CAB) is an investigational integrase strand transfer inhibitor (INSTI) analog of dolutegravir (DTG) that is very potent (50% inhibitory concentration (IC_50_) of about 0.22 nM) and active against various clades of HIV-1^3,4^. The unique physicochemical and pharmacokinetic properties of CAB have permitted its formulation as a long-acting injectable (CAB LA) amenable for dosing every 2–3 months, making CAB LA an attractive alternative to daily oral PrEP regimens^5–7^. Preclinical and phase 2a/b studies in humans have supported the clinical development of CAB LA as PrEP in individuals at high risk of HIV infection. In macaques, CAB LA administered at human therapeutic doses provided significant protection against, rectal, vaginal, penile, and intravenous virus exposures^8–12^. In Phase 2a/b studies, CAB LA was generally safe and well tolerated with injection site reactions that were mild or moderate as the most common adverse events^4,13^. Two large randomized clinical trials (HPTN 083 and 084) are currently evaluating the safety and efficacy of CAB LA for PrEP in men who have sex with men, transgender people, and women.

Although the long plasma half-life of CAB LA is an attractive attribute for PrEP, it also raises concerns about drug resistance emergence if someone becomes infected with HIV or if PrEP is initiated during undiagnosed acute infection. In the ECLAIR trial, CAB was still detectable 52 weeks after the last injection in 14% of subjects^13^. Such a long pharmacologic drug tail can conceivably create opportunities for rapid resistance selection if drug levels are not sufficiently high to prevent infection but are high enough to select for drug-resistant viruses. An additional concern is the lack of information on the mutations selected with CAB in vivo and their potential impact on resistance to other INSTIs, as only two cases of CAB resistance have been reported in HIV-infected patients treated with CAB, both mediated by integrase mutation Q148R^14,15^. It is therefore important to understand risk and mechanisms of CAB resistance arising from the use of CAB LA for PrEP.

To address these questions we modeled in macaques a scenario of CAB LA PrEP initiation during undiagnosed acute HIV infection. We selected a worst-case situation that in early FTC/TDF PrEP trials was commonly associated with emergent resistance^16^. Our modeling approach included infection of macaques with a chimeric simian/human immunodeficiency virus (SHIV) containing the SIV mac239 integrase, followed by CAB-LA treatment initiation after confirmed infection and prior to seroconversion. SIV and HIV share similar integrase resistance pathways in vitro, and the resistance profiles of HIV and SIV integrase mutants is also similar^17–19^. We show selection of integrase mutations G118R, G140R, and E92Q/G with CAB-LA, and demonstrate phenotypic resistance to CAB and cross-resistance to all licensed integrase inhibitors including raltegravir (RAL), elvitegravir (EVG), DTG, and bictegravir (BIC). Our findings highlight the need for appropriate HIV testing algorithms before CAB-LA PrEP initiation, but also support repeat serologic testing shortly after CAB-LA to exclude acute infection and minimize risks of resistance. Data also indicate a potential need to cover the pharmacological drug tail with other prevention modalities during periods of continued HIV infection risk.

## Results

### CAB LA treatment and antiviral activity in macaques

**Fig. 1:**
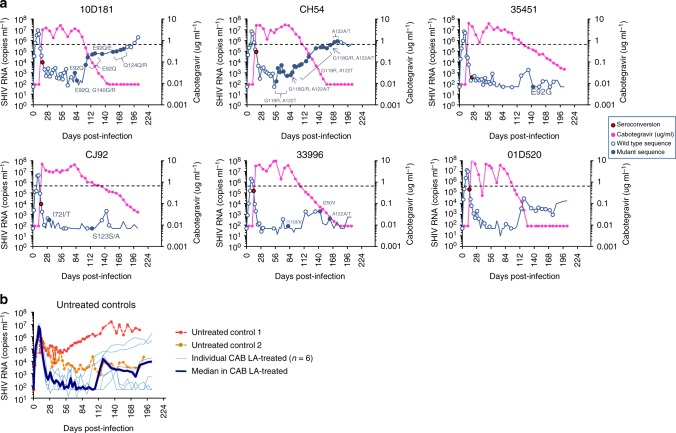
Acute SHIV infection dynamics and concentrations of cabotegravir in plasma. **a** Plasma SHIV RNA levels (blue line) and cabotegravir concentrations (magenta line) in the six macaques treated with cabotegravir long-acting (CAB LA). CAB LA injections were given at days 11, 39, and 67. Macaques seroconverted at days 14–25, always after the first CAB LA injection at day 11. The 4xPA-IC_90_ value (0.664 μg/ml) is shown with a horizontal dotted line. Red circles denote the day of seroconversion. Open blue circles are wild type (WT) integrase sequences and close blue circles are time points with integrase mutations. Time 0 denotes the day of intravenous SHIV inoculation. **b** Plasma SHIV RNA levels in two untreated controls are compared with the median levels observed in the six CAB LA-treated animals. Individual CAB-LA-treated animals are also shown in light blue. Source data are provided as a Source Data file

We infected 8 rhesus macaques intravenously with RT-SHIV and initiated treatment with CAB LA in six of the animals 11 days after infection. The two remaining animals were used as controls. Since the half-life of CAB LA in macaques is shorter than in humans, we administered two subsequent CAB LA injections every 4 weeks (days 39 and 67) to sustain plasma CAB concentrations above four times the protein-adjusted IC_90_ (4xPA-IC_90_; 0.664 μg ml^−1^) for several months and mimic a single 600–800 mg injection in humans. This dosing schedule was effective in maintaining drug concentrations within human therapeutic levels for about 4 months (median = 106 days; range = 95–127) (Fig. 1). After the last injection on day 67, the concentrations of CAB in plasma slowly declined with a half-life of 10.7 days, and were below the limit of detection of the assay in four of the six animals after 115–176 days. The remaining two animals had detectable CAB in plasma at the last measurement on day 204.

Figure 1 shows the kinetics of virus replication seen before and during the treatment and drug washout phase. All animals had detectable SHIV RNA in plasma two days after virus inoculation (median = 4.1 log_10_ copies ml^−1^, range = 2.9–4.7) with peak levels achieved at day 9 (median = 6.7 log_10_ copies ml^−1^; range = 6.3–7.1). Seroconversion was seen between days 14 and 25. Administration of CAB LA at day 11 was associated with a reduction in plasma viremia from a median of 6.6 (6.1–7.0) log_10_ RNA copies ml^−1^ to a median of 2.5 (undetectable—3.3) log_10_ RNA copies ml^−1^ at day 39 (day of the second CAB LA injection) and a median of 1.9 (undetectable—3.7) log_10_ RNA copies ml^−1^ at day 67 (day of the third CAB LA injection). The suppression of virus replication was maintained for an additional 4–5 weeks after the last CAB LA injection at day 67, with RNA levels gradually increasing as the concentrations of CAB fell below the 4xPA-IC_90_ value. Viremias in the controls fluctuated between 3 and 5 log_10_ RNA copies ml^−1^ as is generally seen with this RT-SHIV isolate in rhesus macaques (Fig. 1). Overall, our dosing strategy effectively modeled humans treated with 600–800 mg of CAB and was able to suppress virus replication.

### Virus shedding and CAB concentrations in mucosal fluids

**Fig. 2:**
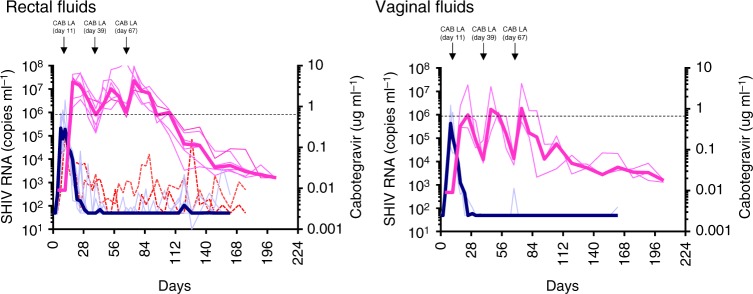
Virus shedding and cabotegravir concentrations in rectal and vaginal fluids. The dark magenta line represents the median concentration of cabotegravir and light magenta lines are the individual animals. The dark blue line represents median SHIV RNA levels seen in treated macaques and light blue lines are the individual animals. The two dotted red lines denote SHIV RNA levels in two male untreated controls. The horizontal dotted line indicates the 4xPA-IC_90_ value (0.664  μg/ml). The three cabotegravir long-acting (CAB LA) injections are shown on top of the figure. Source data are provided as a Source Data file

SHIV RNA and CAB concentrations were also measured in rectal and vaginal fluids. In rectal fluids, the concentrations of CAB were similar to those seen in plasma and remained above the 4xPA-IC_90_ value for a median of 95 days. SHIV RNA levels in rectal fluids were generally low or undetectable during both the treatment period and drug washout phase, as opposed to the two untreated controls that had more consistent virus shedding (Fig. 2). In contrast to rectal fluids, the concentrations of CAB in vaginal fluids were well below the 4xPA-IC_90_ value during the entire treatment period. Despite the low CAB concentrations in vaginal fluids, SHIV RNA was mostly undetectable.

### Integrase mutations in plasma and mucosal fluids

Emergence of integrase mutations was monitored in viruses from plasma and rectal and vaginal fluids. We attempted to amplify the entire integrase region in all the available weekly specimens collected from each animal, although the low viremias seen during treatment with CAB LA limited our ability to generate sequences. Overall, we obtained a median of 25 integrase sequences from plasma per animal, and six and eight sequences from rectal and vaginal fluids, respectively.

The list of integrase mutations identified in plasma from CAB LA-treated animals is shown in Table 1. Macaque 10D181 acquired the E92Q mutation at day 81. At day 85, E92Q was transiently associated with a G140R mutation occurring at a 45% frequency. E92Q was seen in 100% of the virus population during the entire treatment period and the pharmacological drug tail until day 127. The frequency of E92Q declined to 66% at day 134. In this animal, an additional Q124R substitution (frequency ranging between <20 and 100%) was seen between days 155 and 183 when CAB concentrations in plasma were already undetectable. Macaque CH54 acquired both G118R and A122T at day 57. These two mutations represented 100% of the virus population until day 155, with the exception of day 78 when they were seen as 56–59% mixtures with wild type (WT). The frequency of G118R declined to 27% at day 162 and was undetectable between days 170–176. A122T was detectable between days 162 and 176 at frequencies that ranged between 39 and 67% (Table 1).

**Table 1:** Integrase mutations selected in plasma during CAB LA treatment

Macaque ID	Day	Plasma
10D181	81	E92Q
	85	E92Q, G140G/R
	102–127	E92Q
	134	E92E/Q
	155–183	Q124Q/R
CH54	57–71	G118R, A122T
	78	G118G/R, A122A/T
	85–155	G118R, A122T
	162	G118G/R, A122A/T
	170–176	A122A/T
35451	143	E92G
CJ92	32	I72I/T
	127	S123S/A
33996	81	I110I/V
	143	I250V
	162	A122A/T
01D520		NONE

The only mutation identified in macaque 35451 was E92G at day 143 (100% frequency). Macaque CJ92 acquired two substitutions at position 72 and 123 although these mutations were transient and only observed as mixtures (52% I72T at day 32 and 41% S123A at day 127). The I250V and I110I changes seen in macaque 33996 were also identified in one of the untreated animals (macaque 34319, Supplementary Table 1) and are polymorphisms. In treated macaque 33996, A122T was also observed at day 162 (37% frequency) when plasma CAB concentrations were closer to the limit of detection of the assay. No mutations were identified in macaque 01D520 **(**Table 1**)**.

**Fig. 3:**
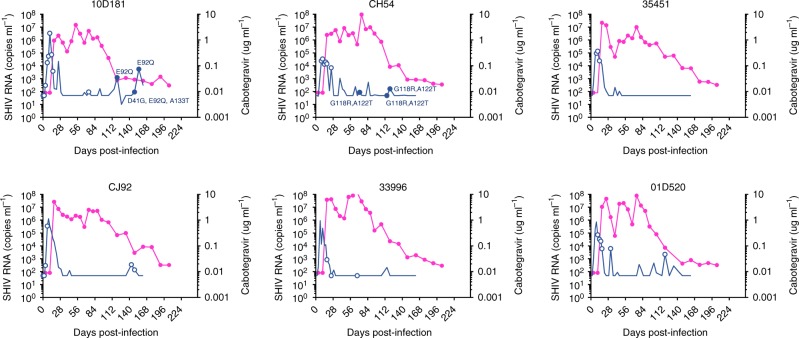
Integrase mutations identified in viruses from rectal fluids. The magenta line indicates the concentrations of cabotegravir and the blue line SHIV RNA levels. Circles denote time points that were successfully amplified and genotyped. Closed circles denote time points with integrase mutations, which are also indicated. Source data are provided as a Source Data file

**Fig. 4:**
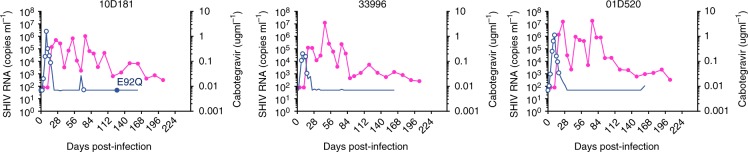
Integrase mutations identified in viruses from vaginal fluids. The magenta line indicates the concentrations of cabotegravir and the blue line SHIV RNA levels. Circles denote time points that were successfully amplified and genotyped. Closed circles denote time points with integrase mutations, which are also indicated. Source data are provided as a Source Data file

The dynamics of virus shedding and integrase mutations in rectal and vaginal fluids are shown in Figs. 3 and 4, respectively. Macaque 10D181 had the E92Q substitution in viruses from rectal fluids at days 120–155 in addition to two polymorphisms at positions 41 and 133 (Fig. 3). E92Q was also detected in vaginal fluids collected from this animal (Fig. 4). Likewise, the G118R and A122T substitutions identified in plasma sequences from macaque CH54 were also detected in rectal fluids between days 71 and 120. All mutations represented 100% of the viral population. No other mutations were seen in viruses from rectal or vaginal fluids in this or any other animal in the few time points that were successfully amplified and sequenced. Supplementary Table 1 shows that the only two integrase mutations identified in the two control macaques were polymorphisms I250V and I110V. Overall, these results document selection of integrase mutations in plasma, rectal and vaginal fluids during CAB LA treatment.

### Effect of integrase mutations on replication and resistance

To investigate the impact of integrase mutations selected in vivo on resistance to CAB and cross-resistance to other integrase inhibitors, we introduced each amino acid change in the SIV integrase and evaluated their effect on susceptibility to CAB, RAL, EVG, DTG, and BIC. Table 2 shows the EC_50_ values for each integrase inhibitor and the changes in susceptibility relative to WT. Of all the mutations identified in vivo, E92Q, E92G, G118R, and G140R were associated with resistance to INSTI. E92Q conferred 2.5-fold resistance to CAB, 3.6-fold resistance to RAL, 7.8-fold resistance to EVG, 1.5-fold resistance to DTG, and 2.0-fold resistance to BIC. Likewise, E92G reduced the susceptibility to CAB by 3.5-fold and also to RAL (2.8-fold), EVG (3.7-fold), DTG (2.8-fold), and BIC (4.3-fold). The G118R and G140R mutations conferred 345- to 1000-fold resistance to all five integrase inhibitors, either alone or in association with A122T or E92Q, respectively (Table 2). Overall, these findings demonstrate that G118R, G140R, and E92Q/G selected during CAB LA treatment are associated with resistance to CAB and cross-resistance to other integrase inhibitors.

**Table 2:** Susceptibility to integrase inhibitors and replicative capacity of site-directed mutants containing integrase mutations

	EFV^a^	RAL	EVG	CAB	DTG	BIC	Replicative capacity (%)^b^
WT	0.67 (1)	2.90 (1)	1.24 (1)	1.19 (1)	1.00 (1)	1.00 (1)	100
E92Q	0.54 (0.8)	10.48 (3.6)	9.67 (7.8)	2.93 (2.5)	1.52 (1.5)	1.97 (2.0)	7.7
G140R	0.59 (0.9)	>1000 (>345)	>1000 (>807)	>1000 (>840)	>1000 (>1000)	>1000 (>1000)	1.3
E92Q/G140R	1.28 (1.9)	>1000 (>345)	>1000 (>807)	>1000 (>840)	>1000 (>1000)	>1000 (>1000)	1.3
Q124R	0.70 (1.0)	4.60 (1.6)	1.59 (1.3)	1.22 (1.0)	1.62 (1.6)	1.49 (1.5)	219
A122T	0.77 (1.2)	4.13 (1.4)	1.15 (0.9)	1.51 (1.3)	1.42 (1.4)	2.10 (2.1)	26.9
G118R	1.01 (1.5)	>1000 (>345)	>1000 (>807)	>1000 (>840)	>1000 (>1000)	>1000 (>1000)	2.3
G118R/A122T	0.72 (1.1)	>1000 (>345)	>1000 (>807)	>1000 (>840)	>1000 (>1000)	>1000 (>1000)	2.2
E92G	1.01 (1.5)	8.07 (2.8)	4.53 (3.7)	4.20 (3.5)	2.78 (2.8)	4.29 (4.3)	7.5
I72T	0.60 (0.9)	2.73 (0.9)	1.17 (0.9)	0.96 (0.8)	1.14 (1.1)	1.51 (1.5)	124
I110V	0.79 (1.2)	3.80 (1.3)	1.55 (1.3)	1.27 (1.1)	1.52 (1.5)	1.78 (1.8)	111.1
I250V	0.67 (1.0)	4.00 (1.4)	1.45 (1.2)	1.04 (0.9)	0.92 (0.9)	1.56 (1.6)	83.7

Values in parenthesis represent fold-change relative to wild type integrase

*EFV* efavirenz, *RAL* raltegravir, *EVG* elvitegravir, *CAB* cabotegravir, *DTG* dolutegravir, *BIC* bictegravir

^a^EC_50_ value in nM

^b^Percentage of wild type

We also evaluated the impact of mutations on replication capacity (Table 2). The replication capacity of mutants containing G118R alone or in combination with A122T was 2.2–2.3% of that seen in WT SHIV. Likewise, the replication capacity of mutants containing G140R with or without E92Q was 1.3%. The E92Q and E92G mutations alone reduced replication capacity to 7.5–7.7% of that seen in WT.

## Discussion

We investigated risks of drug resistance emergence associated with initiating CAB LA for PrEP during undiagnosed acute HIV infection. We used a macaque modeling approach that consisted of CAB LA treatment initiation in seronegative animals that were SHIV RNA positive. We selected CAB LA doses that were sufficient to maintain plasma CAB concentrations above 4xPA-IC_90_ and within human therapeutic levels for 3–4 months. Under these conditions of prolonged exposure to CAB monotherapy, three of the six animals selected for mutations in the integrase that conferred phenotypic resistance to CAB and cross-resistance to other INSTIs. The selection of resistance may have been facilitated by the high acute viremias and prolonged period of monotherapy. These findings are in line with observations from early PrEP trials with FTC/TDF, where most of the cases of drug resistance were seen among participants who initiated PrEP during unrecognized acute HIV infection^16^. Our findings in a macaque model of CAB LA PrEP reiterate the importance of PrEP recommendations and HIV testing algorithms that detect acute infection to minimize PrEP initiation during undiagnosed HIV infection. These should include antigen/antibody combo assays and generic nucleic acid tests.

We document selection of G118R, G140R, and E92Q/G, all of which have been associated with resistance to INSTIs in HIV-1 and in some instances SIV. G118R is a rare non-polymorphic mutation that can cause 5–20-fold resistance to INSTIs in HIV and also resistance to DTG, RAL and EVG in SIV^20^. We found that G118R conferred 345–1000-fold resistance to all five INSTIs. The E92Q mutation previously characterized as a mutation conferring resistance to EVG in SIV and HIV^17,21^ was also associated with 2.5-fold resistance to CAB. Likewise, E92G, a rare non-polymorphic mutation that in HIV confers resistance to EVG^22,23^, was associated with reduced susceptibility to CAB (3.5-fold) and EVG (3.7-fold). The impact of G140R in HIV is not known although other non-polymorphic mutations at position 140 including G140S/A/C are associated with 10-100-fold reduced susceptibility of HIV to RAL, EVG, and DTG^24–26^. We show here that, in SIV, G140R confers 345 to >1000-fold resistance to all five INSTIs, thus expanding the resistance profile associated with changes at position 140. Overall, these results demonstrate intermediate to high-level resistance to CAB and other INSTIs due to G118R, G140R and E92Q/G. Our results also expand the list of mutations associated with CAB resistance in vivo. In humans, treatment with CAB or CAB LA has been associated with rare instances of selection of Q148R^14,15^. It will be important to see if the G118R, G140R and E92Q/G mutations selected in SIV in vivo will also be selected in patients failing CAB-containing regimens, and if these mutations will confer similar levels of drug resistance in HIV. We also found that G118R, E92Q, E92G, and G140R were associated with marked reductions in viral replicative capacity, a finding that is line with that seen in HIV-1^23,27^.

Current dosing strategies of CAB LA for PrEP in humans is 600 mg given every 2 months designed to maintain plasma levels above 4xPA-IC_90_ values, the CAB concentration achieved in our macaques following monthly CAB LA injection. Under this clinical CAB exposure we note that resistance was detected as early as day 57 in one animal, and at day 81 and 143 in two other animals. Therefore, the kinetics data in this macaque model imply that clinical CAB drug resistance could emerge within 2 months before a second CAB LA injection is given. This scenario differs from that of other PrEP drugs such as FTC, which can rapidly select for the M184V/I mutation within days or weeks^16^. The slow dynamics of CAB resistance emergence and the finding that all the macaques seroconverted before the appearance of resistance, suggest that HIV testing 1 month after CAB LA initiation may help diagnose early infection and allow for treatment with combination therapy to prevent resistance emergence. However, it is possible that in our macaque model resistance might have been facilitated by the use of high dose of infectious virus administered intravenously, and that in humans infected with single or few genetic variants resistance might take even longer to emerge. Additional studies with larger number of animals and different virus doses or routes of exposure may help address these questions.

While virus shedding was reduced among CAB-treated animals, the finding of E92Q and G118R in viruses from rectal fluids was noteworthy as it suggested that CAB concentrations in the rectal mucosa were sufficient to select and/or maintain drug-resistant viruses. These findings were consistent with CAB concentrations in rectal fluids that were above 4xPA-IC_90_, and raise important concerns about potential secondary transmission of these mutants, particularly since E92Q and G118R were able to persist in rectal fluids for 1–2 months. Although the in vitro replication capacity mutants containing E92Q or G118R was markedly reduced, their transmission fitness is not known as it is also unknown if their transmission potential may be increased if viremias are high as it is the case of acute HIV infection. Unfortunately, SHIV RNA was difficult to amplify from vaginal fluids, although we found E92Q in the only female animal that had detectable drug-resistant viruses in plasma.

Our study design does not directly inform on risk of resistance emergence during the drug tail after a last CAB LA injection. Reasons for this are the differences in half-life of CAB LA between macaques (11 days) and humans (25–54 days), and a shorter exposure to drug concentrations below 1xPA-IC_90_ threshold, which in preclinical models has been associated with breakthrough SHIV infections^8^. However, the maintenance of G118R and E92G in two of the macaques when CAB levels were low or undetectable suggests a risk of resistance selection during the CAB drug tail. An instance of infection and subsequent selection of drug-resistant viruses was reported in a seroconverter that received a long-acting formulation of rilpivirine 84 days earlier^28^. Pending definitive clinical data, our findings with CAB LA indicate a potential need to cover the pharmacological drug tail with other prevention modalities during periods of continued HIV infection risk in order to minimize infection and selection of drug resistance.

In summary, we show in a macaque model of PrEP initiation during acute HIV infection that mutations conferring resistance to CAB can be frequently selected and maintained for several months. Selection of mutations is concerning as they can potentially compromise future treatment with CAB and other integrase inhibitors, although this last point requires further confirmation in HIV-infected patients. Maintenance of mutations and detection of resistant viruses in mucosal fluids also raise concerns regarding potential secondary transmission of these viruses, particularly due to the high viremias generally seen during acute HIV infection. Our results emphasize the need for appropriate HIV testing strategies for detecting acute infection before initiating CAB LA for PrEP. The finding that the macaques seroconverted long before resistance selection also suggests that repeat serologic testing performed one month after the first CAB LA injection may allow for early HIV diagnosis and enable the initiation of combination therapy to minimize risks of resistance emergence.

## Methods

### Animal care

All the animal procedures performed in this study were reviewed and approved by the Institutional Animal Care and Use Committee (IACUC) of the Centers for Disease Control and Prevention (CDC). Procedures complied with all relevant ethical regulations for animal testing and research. Macaques were housed under the full care of CDC veterinarians in accordance with the standards incorporated in the Guide for the Care and Use of Laboratory Animals (National Research Council of the National Academies, 2010). SHIV infected macaques were humanely euthanized in accordance with the American Veterinary Medical Association Guidelines on Euthanasia, 2013.

### Drug and virus stock

CAB LA (200 mg/mL injectable stock aqueous suspension) was given at a dose of 50 mg kg^−1^ under anesthesia by intramuscular injection into the quadriceps. Volumes greater than 1.0 mL were divided into two injection sites. The RT-SHIV isolate used for this study is a chimeric SIVmac239 that contains the RT of HIV-1HXBc2, and was obtained from the NIH AIDS Research & Reference Reagent Program. RT-SHIV contains a T to C substitution at position 8 of the SIV tRNA primer binding site that improves replication^29–31^.

### Infection of macaques with RT-SHIV and CAB LA treatment

Six Indian rhesus macaques were infected intravenously with 1 ml of cell-free virus (10^3.3^ TCID_50_ mL^−1^) and received 50 mg/kg of CAB LA intramuscularly after confirmed infection but prior to seroconversion (measured using a synthetic-peptide EIA; BioRad, Genetic Systems HIV-1/HIV-2, Redmond, WA). Animals received two subsequent CAB LA injections 4 weeks apart to sustain plasma drug levels above four times the protein-adjusted IC_90_ (4xPA-IC_90_) and model humans treated with 600 mg of CAB LA every 8 weeks^8,11^. Two untreated rhesus macaques were used as controls.

Blood and rectal and vaginal fluids were collected once or twice a week during the treatment period and drug washout phase to monitor virus replication, drug concentrations, and emergence of integrase mutations. Rectal and vaginal fluids were collected in Weck-Cel Surgical Spears (Medtronic Ophthalmic, Jacksonville, FL)^32^. SHIV viremia and mucosal virus shedding was monitored using an RT-PCR assay with a detection limit of 50 copies ml^−1^ ^32^. CAB concentrations were determined by Covance Laboratories Inc (Indianapolis, IN) using a validated liquid chromatography–mass spectrometry method with a lower limit of quantification of 10 ng ml^−1^ ^11^.

### Sequence analysis

SIV integrase was amplified by standard RT nested PCR using outer integrase primers HXB2RT-F1 and MAC239VIF-R1, and inner integrase primers HXB2RT-F2 and MAC239VIF-R2^33^. SHIV RNA was extracted from 1 ml of plasma or two rectal/vaginal wicks using the QIAGEN QIAamp Viral RNA Mini Kit (Qiagen) and resuspended in 50 ul of buffer. The RT reaction was done at 50 C for 60 min, 55 C for 60 min, and 70 C for 15 min using 10 μl of extracted RNA. Based on SHIV RNA copies in plasma and rectal and vaginal fluids, we amplified a median of 457 (<10–173,161), 1740 (<10–675,253) and 4508 (<10–511,809) viral genome copies, respectively. Cycling parameters for the outer PCR were 94 C for 2 min followed by 35 cycles of 94 C for 15 s, 54 C for 30 s, and 68 C for 90 s, with a final extension of 10 min at 72 C. Conditions for the inner PCR were 94 C for 2 min followed by 35 cycles of 94 C for 15 s, 51 C for 30 s, 68 C for 90 s, with a final extension of 10 min at 72 C. Bulk sequences from purified PCR products were generated using an AB 3130xl or 3730xl Genetic Analyzer (Life Technologies) and primers HXB2RT-SEQ1, SIVINT-SEQ3, SIVINT-SEQ4, SIVINT-SEQ5, and SIVINT-SEQ6. A complete list of primers and their sequences is shown in Supplementary Table 2. We generated a total of 51 full-length and 123 partial (amino acids 36–270) integrase sequences. Mutation frequencies were estimated on the bases of the relative peak heights obtained from electropherograms and quantified using the QSVAnalyzer tool (http://dna.leeds.ac.uk/qsv/)^34^. We used a detection frequency lower limit of 20%.

### Phenotypic testing

The SIV integrase resistance testing vector (IN RTV) used in this study was built by modifying the PhenoSense IN RTV containing a luciferase report gene^24^. Specifically, the entire HIV IN coding region was replaced with SIV IN sequence. Single E92G/Q, G140R, Q124R, A122T, G118R, I72T, I110V, and I250V or double E92Q + G140R and G118R + A122T mutations were introduced into the SIV IN region using site-directed mutagenesis (SDM). EFV, RAL, EVG, CAB, DTG, and BIC susceptibilities of the SIV IN and SIV IN SDMs were assessed. Pseudovirus stocks were produced from human embryonic kidney cells (HEK 293 cells obtained from the NIH AIDS Reagent Program) co-transfected with SIV IN RTVs together with amphotropic murine leukemia virus envelope protein expression vector. HEK 293 cells were infected with the pseudovirus stocks in the presence or absence of serial dilutions of IN inhibitors and EFV. Reductions in susceptibility are expressed as fold change (FC) in IC_50_ relative to a WT reference strain.

### Reporting summary

Further information on research design is available in the Nature Research Reporting Summary linked to this article.

## Supplementary information

Supplementary Information

Peer Review File

Reporting Summary

Source Data

## Data Availability

Sequence data supporting the results in Figs. 1, 3, 4 and Table 1 have been deposited in GenBank with accession codes MK127951-MK127989, MK127990-MK128004, and MK128056-MK128124 (partial integrase sequences; amino acids 36–270), and MK128005-MK128055 (full-length integrase sequences). The source data underlying Figs. 1, 2, 3, and 4 are provided as a Source data file.

## References

[1] World Health organization. HIV/AIDS fact sheet World Health organization. http://www.who.int/mediacentre/factsheets/fs360/en/ (2017).

[2] Riddell J, Amico KR, Mayer KH (2018). HIV preexposure prophylaxis: a review. JAMA.

[3] Karmon SL, Mohri H, Spreen W, Markowitz M (2015). GSK1265744 demonstrates robust in vitro activity against various clades of HIV-1. J. Acquir Immune Defic. Syndr..

[4] McPherson TD, Sobieszczyk ME, Markowitz M (2018). Cabotegravir in the treatment and prevention of human immunodeficiency virus-1. Expert Opin. Invest. Drugs.

[5] Trezza C, Ford SL, Spreen W, Pan R, Piscitelli S (2015). Formulation and pharmacology of long-acting cabotegravir. Curr. Opin. HIV Aids..

[6] Spreen W (2014). Pharmacokinetics, safety, and tolerability with repeat doses of GSK1265744 and rilpivirine (TMC278) long-acting nanosuspensions in healthy adults. J. Acquir Immune Defic. Syndr..

[7] Spreen WR, Margolis DA, Pottage JC (2013). Long-acting injectable antiretrovirals for HIV treatment and prevention. Curr. Opin. HIV Aids..

[8] Andrews CD (2014). Long-acting integrase inhibitor protects macaques from intrarectal simian/human immunodeficiency virus. Science.

[9] Andrews CD (2015). A long-acting integrase inhibitor protects female macaques from repeated high-dose intravaginal SHIV challenge. Sci. Transl. Med..

[10] Andrews CD (2017). Cabotegravir long acting injection protects macaques against intravenous challenge with SIVmac251. AIDS.

[11] Radzio J (2015). The long-acting integrase inhibitor GSK744 protects macaques from repeated intravaginal SHIV challenge. Sci. Trans. Med..

[12] Dobard C (2018). Long-acting cabotegravir protects macaques against repeated penile SHIV exposures. Top. Antivir. Med..

[13] Markowitz M (2017). Safety and tolerability of long-acting cabotegravir injections in HIV-uninfected men (ECLAIR): a multicentre, double-blind, randomised, placebo-controlled, phase 2a trial. Lancet Hiv..

[14] Margolis DA (2017). Long-acting intramuscular cabotegravir and rilpivirine in adults with HIV-1 infection (LATTE-2): 96-week results of a randomised, open-label, phase 2b, non-inferiority trial. Lancet.

[15] Margolis, D. A.et al. LAI116482 Study Team. Cabotegravir plus rilpivirine, once a day, after induction with cabotegravir plus nucleoside reverse transcriptase inhibitors in antiretroviral-naive adults with HIV-1 infection (LATTE): a randomised, phase 2b, dose-ranging trial. *Lancet**Infect*. *Dis.* **15**, 1145–1155 (2015). 10.1016/S1473-3099(15)00152-8 26201299

[16] Parikh UM, Mellors JW (2016). Should we fear resistance from tenofovir/emtricitabine preexposure prophylaxis?. Curr. Opin. HIV AIDS.

[17] Hassounah SA (2015). Characterization of the drug resistance profiles of integrase strand transfer inhibitors in simianimmunodeficiency virus SIVmac239. J. Virol..

[18] Wares M, Hassounah S, Mesplède T, Sandstrom PA, Wainberg MA (2015). Simian-tropic HIV as a model to study drug resistance against integrase inhibitors. Antimicrob. Agents Chemother..

[19] Lewis MG (2010). Response of a simian immunodeficiency virus (SIVmac251) to raltegravir: a basis for a new treatment for simian AIDS and an animal model for studying lentiviral persistence during antiretroviral therapy. Retrovirology.

[20] Hassounah SA (2014). Effect of HIV-1 integrase resistance mutations when introduced into SIVmac239 on susceptibility to integrase strand transfer inhibitors. J. Virol..

[21] Kobayashi M (2011). In vitro antiretroviral properties of S/GSK1349572, a next generation HIV integrase inhibitor. Antimicrob. Agents Chemother..

[22] Margot NA (2012). In vitro resistance selections using elvitegravir, raltegravir, and two metabolites of elvitegravir M1 and M4. Antivir. Res..

[23] Abram ME (2013). Impact of primary elvitegravir resistance-associated mutations in HIV-1 integrase on drug susceptibility and viral replication fitness. Antimicrob. Agents Chemother..

[24] Fransen S (2009). Loss of raltegravir susceptibility by human immunodeficiency virus type 1 is conferred via multiple nonoverlapping genetic pathways. J. Virol..

[25] Canducci F (2011). Cross-resistance profile of the novel integrase inhibitor Dolutegravir (S/GSK1349572) using clonal viral variants selected in patients failing raltegravir. J. Infect. Dis..

[26] Underwood MR (2012). The activity of the integrase inhibitor dolutegravir against HIV-1 variants isolated from raltegravir-treated adults. J. Acquir. Immune Defic. Syndr..

[27] Quashie PK (2015). Differential effects of the G118R, H51Y, and E138K resistance substitutions in different subtypes of HIV integrase. J. Virol..

[28] Penrose KJ (2016). Selection of rilpivirine-resistant HIV-1 in a seroconverter from the SSAT 040 trial who received the 300-mg dose of long-acting rilpivirine (TMC278LA). J. Infect. Dis..

[29] Soderberg K (2002). A nucleotide substitution in the tRNA(Lys) primer binding site dramatically increases replication of recombinant simian immunodeficiency virus containing a human immunodeficiency virus type 1 reverse transcriptase. J. Virol..

[30] North TW (2005). Suppression of virus load by highly active antiretroviral therapy in rhesus macaques infected with a recombinant simian immunodeficiency virus containing reverse transcriptase from human immunodeficiency virus type 1. J. Virol..

[31] Uberla K (1995). Animal model for the therapy of acquired immunodeficiency syndrome with reverse transcriptase inhibitors. Proc. Natl Acad. Sci. USA.

[32] Radzio J (2012). Prevention of vaginal SHIV transmission in macaques by a coitally-dependent Truvada regimen. PLoS ONE.

[33] Zheng Q, Ruone S, Switzer WM, Heneine W, Garcia-Lerma JG (2012). Limited SHIV env diversification in macaques failing oral antiretroviral pre-exposure prophylaxis. Retrovirology.

[34] Carr IM (2009). Inferring relative proportions of DNA variants from sequencing electropherograms. Bioinformatics.

