# Supplementary material for: Drug resistance emergence in macaques administered cabotegravir long-acting for pre-exposure prophylaxis during acute SHIV infection
Source: Nat Commun. 2019 May 1;10:2005. doi: 10.1038/s41467-019-10047-w (PMC6494879; doi:10.1038/s41467-019-10047-w)
Supplement: Supplementary file 1 — Supplementary Information [file 41467_2019_10047_MOESM1_ESM.pdf]

**Supplementary information**

# Drug resistance emergence in macaques administered cabotegravir long-acting for pre-exposure prophylaxis during acute SHIV infection

Radzio-Basu et al

**Supplementary Table 1.** List of integrase mutations identified in viruses from plasma and rectal fluids from the two untreated control animals.

| Macaque ID | Day | Mutation |
|---|---|---|
| 34805 (plasma) | 5 | None |
| | 28 | None |
| | 59 | None |
| | 81 | None |
| | 115 | None |
| | 144 | None |
| | 165 | None |
| | 172 | None |
| | 179 | None |
| | 186 | None |
| 34805 (rectal fluids) | 7 | |
| | 26 | |
| | 59 | |
| 34319 (plasma) | 5 | None |
| | 28 | None |
| | 59 | I250I/V |
| | 81 | I250V |
| | 115 | I250V |
| | 144 | I250V |
| | 172 | I250V |
| | 200 | I250V |
| 34319 (rectal fluids) | 14 | None |
| | 28 | None |
| | 172 | I110I/V |

**Supplementary Table 2.** List of primers used to amplify and sequence the SIV integrase

| Primer name | Sequence (5'-3') |
|---|---|
| HXB2RT-F1 | TCT AGC TTT GCA GGA TTC GG |
| MAC239VIF-R1 | CAC TGC ATA AGT ACT GAG CC |
| HXB2RT-F2 | GAA AAG GTC TAT CTG GCA TG |
| MAC239VIF-R2 | CTT CTA AAT GGC TTC CTT CC |
| HXB2RT-SEQ1 | CCA GCA CAC AAA GGA ATT GG |
| SIVINT-SEQ3 | CCT CTA GAT GGG TAC AAT |
| SIVINT-SEQ4 | GAC CCG GTG AGC TAT TGT GG |
| SIVINT-SEQ5 | CGG GTC TAT TAC AGA GAA GG |
| SIVINT-SEQ6 | TTA CTC TGC TGC AGG TCC AC |
