# Supplementary material for: Drug resistance emergence in macaques administered cabotegravir long-acting for pre-exposure prophylaxis during acute SHIV infection
Source: Nat Commun. 2019 May 1;10:2005. doi: 10.1038/s41467-019-10047-w (PMC6494879; doi:10.1038/s41467-019-10047-w)
Supplement: Supplementary file 2 — Peer Review File [file 41467_2019_10047_MOESM2_ESM.pdf]

Reviewers' comments:

Reviewer #1 (Remarks to the Author):

In the manuscript titled "Drug resistance emergence in macaques administered cabotegravir longacting for pre-exposure prophylaxis against SHIV infection", Radzio-Basu et al test whether administration of the long-lasting integrase inhibitor CAB-LA after SHIV infection of rhesus macaques, but prior to seroconversion, results in the development of resistant viral variants. 8 rhesus macaques were inoculated iv with an RT-SHIV and CAB-LA was administered in 6 macaques (there were 2 controls). Similar to studies of FTC and TDF Prep, putative CAB-LA resistance mutations appeared in the plasma 3 of 6 animals. Integrase mutations were also identified in rectal and vaginal fluids of several animals, suggesting that these resistance mutations could be transmitted to other sexual partners. Finally the authors characterize the effects of all the RT-SHIV integrase mutations on the ability of a retrovirus to replicate in the presence of CAB as well as other integrase inhibitors.

The manuscript is generally well-written and communicates important observations as more options of Prep are explored. The manuscript could be significantly improved by the authors by addressing the following points:

1. It is unclear from the manuscript how the integrase mutations are being characterized. Are bulk PCRs being sequenced and frequencies determined by peak height or are clones being sequenced? When an integrase mutation appears, what is it's frequency? How many viral genome copies are being amplified in the integrase RT-PCRs? These questions are integral to the interpretation of the sequencing data.

2. The timing of the appearance of plasma viral integrase mutations should be shown graphically to facilitate comparison with rectal and vaginal data. Again, what are their frequencies in plasma viral populations? And again, how many viral genome copies are being amplified in the RT-PCRs?

3. The discussion should be modified to compare the dynamics and consequences of resistance due to post infection CAB-LA administration to that which occurs during the similar situation with FTC/TDF Prep.

4. The information in the supplemental figure should be incorporated into Figure 1 as the control animal viral loads are important to validate the model.

5. Figures should have visual legends to make interpretation easier. The authors could consider reconfiguring the figures to allow direct comparisons of viral loads and mutations in tissues (plasma, rectal, and vaginal) easier, although this is not required.

Reviewer #2 (Remarks to the Author):

This is an important mechanistic study of the HIV integrase inhibitor cabotegravir (CAB-LA) for the risk of developing drug resistance if CAB-LA PrEP is initiated during undiagnosed acute infection. The study used a macaque model of infection with simian HIV (SHIV) where six macaques infected with RT-SHIV received CAB-LA at clinically relevant doses prior to detection of seroconversion. The study found integrase resistance related sequences of G118R, E92G/Q, or G140R in 3/6 macaques. All these mutations were also associated with reduced viral replicative fitness.

The data presented are very clear and understandable. Figure nicely shows the trends in VL and CAB levels in relation to seroconversion in all mice that were SHIV infected.

A few minor points that could be addressed:

Should make additional notes of limitations of small sample size. Also an IV infection model with a large bolus of modified virus to be highly replicative does not represent the human model of HIV that is often driven by a single or only a few founder viruses. Could the macaque model be prone to higher rates of development of resistance due to the model? Line 211-213 does state it may be different in humans but did not elaborate why.

The earliest a resistance mutation sequence was detected was day 57. While this is could be prior to the 2 month repeat injection visit there does appear to be a window of time to initiate ART prior to emergence of resistance. Would add to summary sentence (line 243-45) that as well as appropriate testing for acute infection, repeat HIV testing prior to the 2 month repeat injection visit may also be helpful to detect early seroconversion and where ART could be initiated prior to development of resistance.

Reviewer #3 (Remarks to the Author):

Radzio-Basu et al. are addressing in their manuscript the issue of drug resistance emergence in macacques receiving long-acting cabotegravir for PrEP.

This is an area of high interest in the prevention field, since long-acting cabotegravir is currently being investigated in two large randomized studies for HIV prevention in both men and women, the results of which will be available in the next couple of years.

The study reported in this manuscript is the first to address this issue in the macaque model which has proven quite reliable to predict what will happen in humans and the authors have a unique expertise in this field.

It raises concerns that resistance not only to cabotegravir but also to all integrase inhibitors might be selected following failure of cabotegravir for PrEP. These data are particularly relevant at a time where WHO is recommending the use of dolutegravir for treatment initiation in all HIV-infected individuals. Indeed, the emergence of integrase resistance in case of cabotegravir failure for PrEP might jeopardize the efficacy of combined antiretroviral therapy for the treatment of HIV-infection.

Futhermore, the presence of resistant isolates in vaginal and rectal fluids also suggest that these isolates could be transmitted to HIV-uninfected individuals, and compromising integrase inhibitors-based regimens.

In addition, these data are the first to assess the emergence of cabotegravir resistance in vivo for PrEP and will be useful to monitor the emergence of these mutations in humans receiving cabotegravir for either treatment or prevention, since cabotegravir is also being investigated for maintenance therapy in combination with rilpivirine in HIV-infected individuals.

There are two major issues in the emergence of HIV drug resistance mutations in people on PrEP:

- The first in the emergence of resistance in people with undiagnosed primary HIV-infection when PrEP with TDF/FTC is started in people with usually very high plasma viral loads receiving therefore suboptimal regimen (dual therapy with two NRTIs). This is the issue addressed in this manuscript since SIV-infected animals started cabotegravir following SIV-infection but before seroconversion at a

time plasma SIV viral loads were very high and cabotegravir plasma drug levels were in the appropriate range. This is the best case scenario to select resistance, as reported in this manuscript and as the authors acknowledge, if used in HIV-negative people for HIV prevention, we must ascertain the absence of HIV-infection before starting cabotegravir long-acting for PrEP.

Interestingly, all animals seroconverted a few days following the first cabotegravir injection, and long before the emergence of resistance mutations (day 57 at the earliest), so one could assume in humans that a serologic assay performed one month after cabotegravir injection could diagnose HIV-infection even if the plasma viral load is suppressed, to start combination antiretroviral therapy and prevent the emergence of integrase resistance which is quite slow to appear in this study. This is quite different from FTC resistance in people starting TDF/FTC during primary HIV-infection where FTc resistance can be detected a few days/weeks after PrEP initiation. Therefore a recommendation of serologic testing one month after cabotegravir initiation may prevent the emergence of integrase resistance and this recommendation could be tested in this animal model. This issue could be addressed in the discussion.

- The second issue is related to the selection of cabotegravir resistance in people starting PrEP while not infected with HIV, but who may experience breakthrough HIV-infection because of lack of adherence and/or insufficient plasma level of cabotegravir. This is another important issue with the use of cabotegravir long acting for PrEP if people do not get their injection at the appropriate time. According to the long half-life of cabotegravir, low drug levels in plasma may be insufficient to protect from HIV-infection and could furthermore select for drug-resistance breakthrough HIV-infection as it has been reported in a patient failing rilpivirine LA (this reference should be added: Selection of Rilpivirine-Resistant HIV-1 in a Seroconverter From the SSAT 040 Trial Who Received the 300-mg Dose of Long-Acting Rilpivirine (TMC278LA). Penrose KJ, Parikh UM, Hamanishi KA, Else L, Back D, Boffito M, Jackson A, Mellors JW.J Infect Dis. 2016 Mar 15;213(6):1013-7).

However this issue is not addressed in this manuscript as the authors acknowledge in the discussion, and further experiments would need to be conducted if one wants to address this issue. Indeed, we have no data at this point to suggest that low plasma level of cabotegravir will select for integrase resistance in case of breakthrough infection due to its high genetic barrier as compared to rilpivirine.

Therefore, the manuscript title should explicitly refer to : Drug resistance emergence in macaques administered cabotegravir LA for PrEP "during acute SIV-infection". Also, the data presented here do not provide information regarding the need to cover the long pharmacokinetic drug tail of cabotegravir for PrEP in someone not already infected with HIV. In the ECLAIR study, the patient who experienced a breakthrough HIV-infection, no integrase resistance was identified. This information should also be provided in this manuscript.

The methods are appropriate and the data generated of high quality.
In Fig.1 The authors would need to show, using closed circles, the time of emergence and nature of resistance mutations, similar to Fig3, and to add the graphs for the two control animals. Also each time point where no integrase mutation was identified should be labeled as WT (wild type) to clearly identify when mutations did emerge.

RESPONSE TO REVIEWERS

Reviewer #1 (Remarks to the Author):

In the manuscript titled "Drug resistance emergence in macaques administered cabotegravir longacting for pre-exposure prophylaxis against SHIV infection", Radzio-Basu et al test whether administration of the long-lasting integrase inhibitor CAB-LA after SHIV infection of rhesus macaques, but prior to seroconversion, results in the development of resistant viral variants. 8 rhesus macaques were inoculated iv with an RT-SHIV and CAB-LA was administered in 6 macaques (there were 2 controls). Similar to studies of FTC and TDF Prep, putative CAB-LA resistance mutations appeared in the plasma 3 of 6 animals. Integrase mutations were also identified in rectal and vaginal fluids of several animals, suggesting that these resistance mutations could be transmitted to other sexual partners. Finally the authors characterize the effects of all the RT-SHIV integrase mutations on the ability of a retrovirus to replicate in the presence of CAB as well as other integrase inhibitors.

The manuscript is generally well-written and communicates important observations as more options of Prep are explored. The manuscript could be significantly improved by the authors by addressing the following points:

1. It is unclear from the manuscript how the integrase mutations are being characterized. Are bulk PCRs being sequenced and frequencies determined by peak height or are clones being sequenced? When an integrase mutation appears, what is it's frequency? How many viral genome copies are being amplified in the integrase RT-PCRs? These questions are integral to the interpretation of the sequencing data.

We clarify in page 15 of the methods section that these are bulk sequences and, based on plasma viremia and the amount of RNA used in the RT-PCR reaction, we estimate that we amplified a median of 387 viral genome copies (page 15 last paragraph). We also indicate in page 16 that the frequencies were determined by peak height calculated using the QSVAnalyzer tool with a detection frequency lower limit of 20%. We now specify the frequencies in pages 9-10.

2. The timing of the appearance of plasma viral integrase mutations should be shown graphically to facilitate comparison with rectal and vaginal data. Again, what are their frequencies in plasma viral populations? And again, how many viral genome copies are being amplified in the RT-PCRs?

As requested, we added this information into Figure 1. Please see also our response above to the comment on mutation frequencies and number of viral genome copies amplified.

3. The discussion should be modified to compare the dynamics and consequences of resistance due to post infection CAB-LA administration to that which occurs during the similar situation with FTC/TDF Prep.

We thank the reviewer for this important comment and include a new paragraph at the end of page 13 that discusses the dynamics of CAB resistance compared to FTC/TDF PrEP, and how the slower dynamics of CAB resistance may provide possible opportunities to avoid resistance emergence.

4. The information in the supplemental figure should be incorporated into Figure 1 as the control animal viral loads are important to validate the model.

As requested, we added the data from the 2 control animals into Figure 1 (panel B) and modified the text on page 7 to indicate this.

5. Figures should have visual legends to make interpretation easier. The authors could consider reconfiguring the figures to allow direct comparisons of viral loads and mutations in tissues (plasma, rectal, and vaginal) easier, although this is not required.

As requested, we added visual legends to aid in the interpretation of the figures. We prefer not to reconfigure the figures by animal ID/tissues to avoid too many panels in the same figure.

Reviewer #2 (Remarks to the Author):

This is an important mechanistic study of the HIV integrase inhibitor cabotegravir (CAB-LA) for the risk of developing drug resistance if CAB-LA PrEP is initiated during undiagnosed acute infection. The study used a macaque model of infection with simian HIV (SHIV) where six macaques infected with RT-SHIV received CAB-LA at clinically relevant doses prior to detection of seroconversion. The study found integrase resistance related sequences of G118R, E92G/Q, or G140R in 3/6 macaques. All these mutations were also associated with reduced viral replicative fitness.

The data presented are very clear and understandable. Figure nicely shows the trends in VL and CAB levels in relation to seroconversion in all mice that were SHIV infected.

A few minor points that could be addressed:

Should make additional notes of limitations of small sample size. Also an IV infection model with a large bolus of modified virus to be highly replicative does not represent the human model of HIV that is often driven by a single or only a few founder viruses. Could the macaque model be prone to higher rates of development of resistance due to the model? Line 211-213 does state it may be different in humans but did not elaborate why.

We added these important points at the end of the first paragraph in page 12.

The earliest a resistance mutation sequence was detected was day 57. While this is could be prior to the 2 month repeat injection visit there does appear to be a window of time to initiate ART prior to emergence of resistance. Would add to summary sentence (line 243-45) that as well as appropriate testing for acute infection, repeat HIV testing prior to the 2 month repeat injection visit may also be helpful to detect early seroconversion and where ART could be initiated prior to development of resistance.

We thank the reviewer for his/her insightful comment and have now included a new paragraph at the end of page 11 addressing this point. We further highlight this point in the last paragraph of the discussion.

Reviewer #3 (Remarks to the Author):

Radzio-Basu et al. are addressing in their manuscript the issue of drug resistance emergence in macacques receiving long-acting cabotegravir for PrEP.

This is an area of high interest in the prevention field, since long-acting cabotegravir is currently being investigated in two large randomized studies for HIV prevention in both men and women, the results of which will be available in the next couple of years.

The study reported in this manuscript is the first to address this issue in the macaque model which has proven quite reliable to predict what will happen in humans and the authors have a unique expertise in this field.

It raises concerns that resistance not only to cabotegravir but also to all integrase inhibitors might be selected following failure of cabotegravir for PrEP. These data are particularly relevant at a time where WHO is recommending the use of dolutegravir for treatment initiation in all HIV-infected individuals. Indeed, the emergence of integrase resistance in case of cabotegravir failure for PrEP might jeopardize the efficacy of combined antiretroviral therapy for the treatment of HIV-infection.

Futhermore, the presence of resistant isolates in vaginal and rectal fluids also suggest that these isolates could be transmitted to HIV-uninfected individuals, and compromising integrase inhibitors-based regimens.

In addition, these data are the first to assess the emergence of cabotegravir resistance in vivo for PrEP and will be useful to monitor the emergence of these mutations in humans receiving cabotegravir for either treatment or prevention, since cabotegravir is also being investigated for maintenance therapy in combination with rilpivirine in HIV-infected individuals.

There are two major issues in the emergence of HIV drug resistance mutations in people on PrEP:

- The first in the emergence of resistance in people with undiagnosed primary HIV-infection when PrEP with TDF/FTC is started in people with usually very high plasma viral loads receiving therefore suboptimal regimen (dual therapy with two NRTIs). This is the issue addressed in this manuscript since SIV-infected animals started cabotegravir following SIV-infection but before seroconversion at a time plasma SIV viral loads were very high and cabotegravir plasma drug levels were in the appropriate range. This is the best case scenario to select resistance, as reported in this manuscript and as the authors acknowledge, if used in HIV-negative people for HIV prevention, we must ascertain the absence of HIV-infection before starting cabotegravir long-acting for PrEP.

Interestingly, all animals seroconverted a few days following the first cabotegravir injection, and long before the emergence of resistance mutations (day 57 at the earliest), so one could assume in humans that a serologic assay performed one month after cabotegravir injection could diagnose HIV-infection even if the plasma viral load is suppressed, to start combination antiretroviral therapy and prevent the emergence of integrase resistance which is quite slow to appear in this study. This is quite different from FTC resistance in people starting TDF/FTC during primary HIV-infection where FTc resistance can be detected a few days/weeks after PrEP initiation. Therefore a recommendation of serologic testing one month after cabotegravir initiation may prevent the emergence of integrase resistance and this recommendation could be tested in this animal model. This issue could be addressed in the discussion.

We thank the reviewer for this insightful comment and have now included a new paragraph at the end of page 11 addressing this topic. We further highlight this point in the last paragraph of the discussion.

- The second issue is related to the selection of cabotegravir resistance in people starting PrEP while not infected with HIV, but who may experience breakthrough HIV-infection because of lack of adherence and/or insufficient plasma level of cabotegravir. This is another important issue with the use of cabotegravir long acting for PrEP if

people do not get their injection at the appropriate time. According to the long half-life of cabotegravir, low drug levels in plasma may be insufficient to protect from HIV-infection and could furthermore select for drug-resistance breakthrough HIV-infection as it has been reported in a patient failing rilpivirine LA (this reference should be added: Selection of Rilpivirine-Resistant HIV-1 in a Seroconverter From the SSAT 040 Trial Who Received the 300-mg Dose of Long-Acting Rilpivirine (TMC278LA). Penrose KJ, Parikh UM, Hamanishi KA, Else L, Back D, Boffito M, Jackson A, Mellors JW.J Infect Dis. 2016 Mar 15;213(6):1013-7).

This reference is mentioned and discussed on page 12-13

However this issue is not addressed in this manuscript as the authors acknowledge in the discussion, and further experiments would need to be conducted if one wants to address this issue. Indeed, we have no data at this point to suggest that low plasma level of cabotegravir will select for integrase resistance in case of breakthrough infection due to its high genetic barrier as compared to rilpivirine.

Therefore, the manuscript title should explicitly refer to : Drug resistance emergence in macaques administered cabotegravir LA for PrEP "during acute SIV-infection". Also, the data presented here do not provide information regarding the need to cover the long pharmacokinetic drug tail of cabotegravir for PrEP in someone not already infected with HIV. In the ECLAIR study, the patient who experienced a breakthrough HIV-infection, no integrase resistance was identified. This information should also be provided in this manuscript.

We modified the title as suggested. We recognize that the current design does not necessarily address the need to cover the drug tail, and indicate that although the findings suggest a potential need, this will require definitive clinical data (page 13, end of first paragraph). We deleted this statement from the abstract to minimize confusion.

The methods are appropriate and the data generated of high quality.

In Fig.1 The authors would need to show, using closed circles, the time of emergence and nature of resistance mutations, similar to Fig3, and to add the graphs for the two control animals. Also each time point where no integrase mutation was identified should be labeled as WT (wild type) to clearly identify when mutations did emerge.

Figure 1 has been modified as requested.

REVIEWERS' COMMENTS:

Reviewer #1 (Remarks to the Author):

The authors have sufficiently addressed this reviewer's comments, except for the following very minor comment:

1. In the materials and methods, the author's should include the viral genome input copy number estimates for the vaginal and rectal RT-PCR reactions used for sequencing alongside those estimates for plasma mutation sequencing.

Thomas Vanderford

Reviewer #2 (Remarks to the Author):

The authors have responded adequately to the reviewers comments.

Reviewer #3 (Remarks to the Author):

The authors have appropriately addressed the reviewers concerns.
One issue that may need to be emphasized in the lack of detection of integrase resistance in the two controls animals in blood, vaginal and rectal secretions. This information could be provided in text, table and Figures so the readers could see how many time points were tested in these two controls animals.

RESPONSE TO REVIEWERS

Reviewer #1 (Remarks to the Author):

The authors have sufficiently addressed this reviewer's comments, except for the following very minor comment:

1. In the materials and methods, the author's should include the viral genome input copy number estimates for the vaginal and rectal RT-PCR reactions used for sequencing alongside those estimates for plasma mutation sequencing.
As requested, we now include input copy estimates from rectal and vaginal fluids in the Methods section (page 16, last paragraph)

Reviewer #3 (Remarks to the Author):

The authors have appropriately addressed the reviewers concerns.
One issue that may need to be emphasized in the lack of detection of integrase resistance in the two controls animals in blood, vaginal and rectal secretions. This information could be provided in text, table and Figures so the readers could see how many time points were tested in these two controls animals.

As requested, we now provide a new Supplementary Table 1 that specifies the time points that were sequenced in plasma and rectal fluids from the 2 untreated controls as well as the integrase mutations identified. These results are also described in page 9 (first paragraph).
